# A qualitative evaluation of the oncologists’, neurologists’, and pain specialists’ views on the management and care of chemotherapy-induced peripheral neuropathy in The Netherlands

**DOI:** 10.1007/s00520-024-08493-4

**Published:** 2024-04-22

**Authors:** F. G. A. M. van Haren, M. A. H. Steegers, K. C. P. Vissers, S. A. S. van den Heuvel

**Affiliations:** 1 Department of Anesthesiology Pain- and Palliative Medicine, Radboudumc, Nijmegen, The Netherlands; 2 Department of Anesthesiology Pain- and Palliative Medicine, AmsterdamUMC, Amsterdam, The Netherlands

**Keywords:** CIPN, Patient care, Standardizing care, Neuropathic pain, Qualitative research

## Abstract

**Purpose:**

In treating cancer, different chemotherapy regimens cause chemotherapy-induced peripheral neuropathy (CIPN). Despite recent international guidelines, a gold standard for diagnosis, treatment, and care is lacking. To identify the current clinical practice and the physicians’ point of view and ideas for improvement, we evaluated CIPN care by interviewing different specialists involved.

**Methods:**

We performed semi-structured, audio-recorded, transcribed, and coded interviews with a purposive sample of oncologists, pain specialists, and neurologists involved in CIPN patients’ care. Data is analyzed by a constant comparative method for content analysis, using ATLAS.ti software. Codes, categories, and themes are extracted, generating common denominators and conclusions.

**Results:**

With oncologists, pain specialists, and neurologists, nine, nine, and eight interviews were taken respectively (including three, two, and two interviews after thematic saturation occurred). While useful preventive measures and predictors are lacking, patient education (e.g., on symptoms and timely reporting) is deemed pivotal, as is low-threshold screening (e.g., anamnesis and questionnaires). Diagnosis focusses on a temporal relationship to chemotherapy, with adjuvant testing (e.g., EMG) used in severe or atypical cases. Symptomatic antineuropathic and topical medication are often prescribed, but personalized and multidimensional care based on individual symptoms and preferences is highly valued. The limited efficacy of existing treatments, and the lack of standardized protocols, interdisciplinary coordination, and awareness among healthcare providers pose significant challenges.

**Conclusion:**

Besides the obvious need for better therapeutic options, and multidisciplinary exploration of patients’ perspectives, a structured and collaborative approach towards diagnosis, treatment, referral, and follow-up, nurtured by improving knowledge and use of existing CIPN guidelines, could enhance care.

**Supplementary Information:**

The online version contains supplementary material available at 10.1007/s00520-024-08493-4.

## Introduction

With advances in the treatment of cancer, the length of survival increases, and more patients live with long-term side effects. This emphasizes the importance of a focus on these side effects as well as quality of life (QoL) besides cancer curation [1–3]. One chronic side effect and major dose-limiting toxicity of chemotherapy treatment (e.g., not only platinum-, taxane, and vinka-alkaloid based, but also newer substances) is chemotherapy-induced peripheral neuropathy (CIPN) [4–7]. It presents as a persistent peripheral, “stocking-and-glove” distributed neuropathy affecting 13–79.2% of patients to a variable extent after 15 months [8–10].

An insurance claim data study including 53 million patients found a relative underrepresentation of the incidence of CIPN as compared to large clinical studies. This is possibly explained by a lack of an evidence-based approach resulting in a failure to report [10].

CIPN may present diagnostic issues (e.g., no golden standard) in its primary identification. Evidence-based proven treatment and follow-up strategies are lacking, although recent American Society of Clinical Oncology (ASCO) [11] and European Society for Medical Oncology (ESMO) [12] guidelines on CIPN provide some framework. Our hypothesis is that standardization of the diagnosis and treatment of CIPN, and implementation of existing guidelines, might be suboptimal but mandatory to improve CIPN care and QoL. In acquiring the best reflection on the majority of CIPN patients and common complaints, and in the context of the Dutch healthcare organization, we interviewed accredited oncologists, neurologists, and pain specialists to gain insight in how different specialists approach CIPN care. We explored their views on healthcare organization and patient specific CIPN care as well as opinions on needed improvements.

## Methods

This explorative qualitative study with semi-structured interviews was performed after approval by the local research ethical committee (METC Amsterdam UMC (VU), The Netherlands, dossier number: 2021.0069).

### Participants

Registered oncologists, neurologists, and pain specialists, involved in the care for patients with CIPN, were invited to participate in this interview study. Written (e-mail) and oral information was provided (practical, on anonymity, on withdrawal) and informed consent for participation and publication was obtained before any actual interview. Recruitment of participants continued until thematic saturation was achieved per specialty, after which at least two more interviews were performed. Per specialty, eight to nine interviews were conducted, with a total of 26.

### Procedure

Data was collected by semi-structured interviews, starting from a topic list as presented in Table 1 and a first general question: “Could you talk about your involvement in the care for patients with CIPN?” As the interview progressed, participants were asked to elaborate on the topics and as new ones arose, topics were added. Questions were open-ended and broad, allowing description of experiences without being overly structured by the guide, asking participants to elaborate on topics in the updated list. Each participant was interviewed once, either through Microsoft Teams (Microsoft, www.microsoft.com) or Zoom Video Conference (Zoom Video Communications, www.zoom.us) to the interviewee’s preference. Researchers FH, BW, and NB are medical doctors (MD) receiving additional training to the conduct of these semi-structured interviews, in addition to their regular extensive communication training.

**Table 1:** Interview guide, giving an overview as well as guidance on topics in the interview

General: 1. In what type of hospital do you work? 2. What is your involvement in CIPN care? 3. Do you use a protocol regarding CIPN in your clinical practice? 4. Is this protocol local, national or international?
Chemotherapy regimen: 5. What information is provided to a patient at the start of chemotherapy? 6. How do you screen for pre-existing peripheral neuropathy? 7. What role do risk factors play? 8. Do you use any preventive measures regarding CIPN?
Diagnosis: 9. Are patients screened for CIPN during chemotherapeutic treatment? 10. How is this screening performed? 11. Who primarily diagnoses a patient with CIPN? 12. How is a CIPN diagnosis made? 13. How do you register CIPN or its symptoms?
Treatment: 14. What is your first course of action when a patient is diagnosed with CIPN? 15. What symptomatic treatments options do you use in CIPN? 16. Are changes ever made to the oncological treatment? If yes, how? 17. Are there any other treatment options that are used for CIPN?
Referral and follow-up: 18. Who monitors the symptoms of CIPN after chemotherapy? 19. Are there any other specialists consulted when diagnosing or treating patients with CIPN? If yes, who and why? 20. What role does a general practitioner play in the care for patients with CIPN?
Limitations and improvements: 21. Are there any limiting factors in the current practice regarding CIPN care? 22. Can you name some areas of improvement regarding this care?

### Data analysis

Interviews were audio-recorded, transcribed verbatim, and entered into Atlas.ti Software v.23.1.1 (http://atlasti.com Atlas.ti Scientific Software Development GmbH, Berlin, Germany). Gestures, pauses, perceived hesitations, and notable changes in tone of voice and emphasis were included as notes in the transcription, after which the video file was deleted, and the audio file saved as part of the electronic case report form. Data collection, coding, and analysis started after a specific interview was completed, so these phases occurred simultaneously as the study progressed. Qualitative data was analyzed by an inductive process, using a constant comparative method for direct content analysis, generating direct information from participants without imposing preconceived categories after open coding and using codes from previous interviews as a starting point for the following [13]. Two researchers periodically discussing findings and discrepancies, referring to the data as needed until complete agreement was reached. Analysis progressed through an iterative process of reducing data into categories and themes.

## Results

### Sample

Interviews were conducted from January 2021 to June 2023. As mentioned, after thematic saturation occurred, at least two additional interviews were conducted to strengthen the conclusion. Accordingly, the number of interviews conducted was 6 + 3 = 9 for medical oncologists, 7 + 2 = 9 for pain specialists, and 6 + 2 = 8 for neurologists. In two additional interviews with oncologists, one new code came up, and a third additional interview was conducted. Interviews lasted 21 to 30, 23 to 50, and 16 to 33 min for oncologists, pain specialists, and neurologists, respectively. The participants’ characteristics are presented in Table 2. Quotes, numbered as [quote N°] in the results text, are displayed in the Electronic Supplementary Material.

**Table 2:**
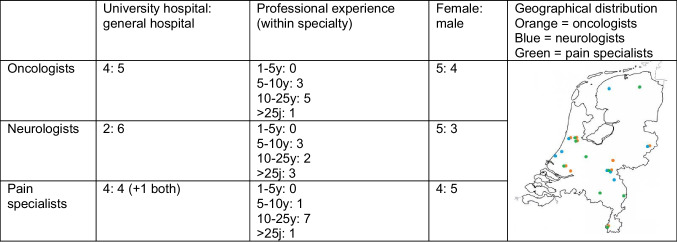
Characteristics of the interviewees

### Medical oncologists

In total, 37 descriptive codes were extracted from the data after coding, divided into nine categories, and subdivided into five themes. A schematic overview of codes, categories, and themes is provided in Table 3.

**Table 3:** Oncologists—codes, categories, and themes extracted from the interview data after coding

Codes	Categories	Themes
Prevention Risk factors Predictors CIPN Prevention: Insufficient evidence	Prevention	Preparation
Informing: Acute peripheral neuropathy Informing: Chronic peripheral neuropathy Informing: Impact CIPN on oncological treatment Informing: Others	Informing the patient	
Screening before treatment Screening during treatment Screening after treatment Screening questionnaires Screening input patient Estimating grade of CIPN	Screening	Assessment
Diagnose: First suspicion Diagnose: Additional actions	Diagnosis	
Symptomatic treatment: Side effects Initiator of therapy Symptomatic pharmacological interventions Effectiveness of symptomatic treatment Symptomatic treatment: Others	Symptomatic treatment	Therapy
Acceptance Oncological decision-making before treatment Oncological decision-making during treatment Oncological decision-making: Treatment setting Oncological decision-making: Multi-disciplinary approach Cause of pre-existing peripheral neuropathy	Oncological decision-making	
Algorithm Registration	Organization	Healthcare system
General practitioner Referral specialists Referral paramedic	Other healthcare providers	
Improvement: More awareness Improvement: Prevention Improvement: Clear guidelines Improvement: Diagnosis and treatment Improvement: Standardization of care	Improvements	Area of improvement

#### Theme 1: Prevention

Lacking evidence of efficacy, preventive measures are rarely used in CIPN, excluding general advice (quotes 1–3). Pre-existing peripheral neuropathy (e.g., diabetic or alcoholic) influences the oncological treatment plan, while risk factors are only a reason for specific actions or referral for some (quotes 4–8). A need for useful predictors is emphasized (quotes 9–10).

In some cases, you stop the treatment and ask yourself: ‘did I stop to early?’ and in other cases when a patient develops symptoms you think: ‘why didn’t I stop earlier?’ (quote 10).

Informing patients and expectation management are deemed vital and are provided orally by the oncologist and specialized nurses supplemented with written information. It includes (uncertainties on) symptom development and “coasting” (increasing symptoms after chemotherapy cessation) (quotes 11–13).

#### Theme 2: Assessment

All oncologists perform a brief (verbal) screening on pre-existing peripheral neuropathy and risk factors before each chemotherapy cycle, sometimes referring to a neurologist when present (quote 14). Reporting CIPN symptoms is actively encouraged, improving follow-up, and thus detecting neurotoxicity earlier (quotes 15–16). For CIPN grading, the Common Toxicity Criteria for Adverse Events (CTCae) is used, most commonly when further assessing symptoms (14) (quotes 17–18, 24–25). Patient-reported outcome measures (PROMs) questionnaires are only used in the context of clinical studies (quotes 19–20). In uncertainty, consulting a neurologist and change in chemotherapy-regimen are considered (quotes 26–28).

Follow-up regarding side effects differs after adjuvant chemotherapy (mainly by the surgeon) and after chemotherapy in metastasized disease (mainly by the oncologist), for patients with severe symptoms of CIPN and in a palliative setting (the oncologist or nurse practitioner) (quotes 21–23).

#### Theme 3: Therapy

Symptomatic treatment is usually started for pain and/or poor sleep quality, using amitriptyline, nortriptyline, pregabalin, and gabapentin, but not duloxetine (quotes 29–31). Because of side effects or insufficient effect, half the patients stop taking this medication in time (quote 34). Non-pharmacological advice is practical and involves foot care, avoidance of painful stimuli (e.g., cold prevention), or support in coping (quotes 35–36).

Reduced initiation doses are a strategy in pre-existing peripheral neuropathy or in a distinct wish to preserve fine motor skills. Complex cases are discussed among colleagues (quotes 37–39). Later dose reduction is usually protocolized, 25% (in neurotoxicity grade ≥ 2) up to 50% (quote 40), and determined by chemotherapy goal (adjuvant or palliative, impact on survival) or pre-existing neuropathies. Side effects are accepted as being (partially) inevitable, and thus must be embedded in the informed consent (quotes 41–45).

#### Theme 4: The healthcare system

Knowledge on existing diagnostic and treatment algorithms is scarce (quote 46). Algorithms are partially embedded in (local) treatment protocols, but do not assist in diagnosing and treating CIPN (quotes 47–49). Information on side effects is individually registered, but no national database collecting risk factors and outcome-related (scientific) data exists (quotes 50–51).

General practitioners contact the oncologist in case of general side effects or CIPN, and its treatment (quote 52). If symptomatic treatment fails or in high grades of neurotoxicity, neurologists, pain specialists, or subspecialized neuro-oncologists are involved (quotes 53–54) as are occupational therapists, physiotherapists, and/or podiatrists for practical advice and support in coping (quotes 55–56).

#### Theme 5: Areas of improvement

Improving awareness and attention for CIPN is regarded essential in improving CIPN care. Therefore, available epidemiological data treatment options should be improved (quotes 57–59).

I think that it can be a good start to gather more information of our own patients so that we can perform more epidemiological studies on Dutch patients. With that we can also create some more awareness I hope. (quote 58).

Also, a certain exposure or experience with chemotherapeutic agents benefits early recognition of CIPN symptoms (quotes 60–61), as well as standardization of diagnosis and treatment (quote 62). New and more effective strategies in the prevention and treatment of CIPN would be welcomed, as well as less (neuro)toxic oncological regiments (quote 63).

### Neurologists

In total, 66 descriptive codes were extracted from the data after coding, divided into 16 categories, and subdivided into six themes. A schematic overview of codes, categories, and themes is provided in Table 4.

**Table 4:** Neurologists—codes, categories, and themes extracted from the interview data after coding

Codes	Categories	Themes
Patient flow	Healthcare organization	The organization of healthcare
Referral structure		
Practice variation		
Care path		
Reimbursement		
Healthcare		
Efficiency		
Neurology	Other specialties	
Anesthesiologist—pain physician		
Oncology		
Multidisciplinary consultation and cooperation	Cooperation and referral	
Referral question		
Referring patients		
Follow-up		
Who starts treatment		
Who does the consultation		
Which patient gets referred		
Chemotherapy	Chemotherapy and cancer related aspects	Risk factors and confounding diseases
Cancer and chemotherapy		
Prevention and chemotherapy dose		
Uncertainty		
Risk factors	Risk factors and differential diagnosis	
Other morbidity		
Other causes of chemotherapy		
Preferred medication	Medication	Treatment (in)possibilities
Medicinal cannabis		
Side effects		
Topical treatment		
Opioids		
Antineuropathic medication		
Treatment	Treatment variables	
Treatment options		
Treatment variability		
Multimodal treatment	Non-medicinal treatment	
Rehabilitation		
Physical therapy		
Guidance (general—psychological—socially)		
Frustration	Frustration	
Social participation	Social participation	Socio-environmental aspects
Emotional well-being	Patient-related multimodal aspects	
Acceptation		
Informing and consulting patients		
Self-sustainability and -direction		
Patient focussed		
Anamnesis	Diagnostic resources	Diagnostic conundrums
Electromyography		
Other (medical) examinations		
Clinical picture		
Blood tests		
Questionnaires		
Neurological examinations		
Diagnostic process	Diagnostic—general	
Diagnosis		
Algorithm for diagnosis		
Incertainty		
Painful (poly)neuropathy	(Course of) symptoms	
Course of complaints		
CIPN symptoms		
Pain		
Chronic symptoms		
Evidence-based medicine	EBM and research	Towards proven and standardized diagnosis and treatment
Drug research		
Scientific research		
Mechanism of CIPN		
Protocols	Protocols and guidelines	
Guidelines		

#### Theme 1: The organization of healthcare

The organization of (multidisciplinary) care depends on local possibilities and patient influx. While sub-specialization and the exchange of ideas is encouraged, the case load is usually spread random. Most are involved through neuro-oncological expertise, within existing neuro-oncological or palliative care meetings and random (ad hoc) consultation, as no specific CIPN meetings exist. Although reimbursement concerns seem minor, opinions differ on the value of the time and effort put into frequent patient visits and additional testing. There is a perception of randomness in policies for referral, diagnosis, and treatment, varying per oncologist, tumor site, chemotherapy regimen, and clinical picture, highlighting the need for structured frameworks (quotes 64–65).

I think variation is large. This does not necessarily have to be wrong; some doctors feel more comfortable starting specific treatments than others. But a framework, some agreement… who will start treatment, which patient needs a neurologist or a pain physician… Yet then, what exactly is the best treatment? To my opinion, even that is not certain. So, something practical: who, what, when? That, I would welcome. (quote 64).

The impression is that the oncologist usually starts CIPN treatment, assuming an expectation of limited additional diagnostic or treatment options on referral.

Because I can imagine that oncologists do not refer patients to us, claiming we do not add much. Which, unfortunately, for some patients, is true. (quote 66).

The neurologists’ added value concerns functional aspects and specific skills in (indicating) specific testing and differential diagnosis, but not analgetic drug prescription specifically. Referral questions regard chemotherapy (e.g., discontinuation, optimalization of concurrent problems, follow-up), diagnosis, or the need for additional testing by EMG, or are study-related, and less frequent to justify dose reduction. Follow-up is done by the specialist prescribing pain medication (quote 67).

#### Theme 2: Risk factors and confounding diseases

After screening for additional risk factors, an EMG is incidentally requested for diagnosis and follow-up, but only in research at baseline. Its predictive value for the evolution and prognosis of CIPN complaints is regarded low. For the exclusion of co-morbidity causing neuropathy, blood tests are often done according to the Dutch neurologists’ guidelines [14], but one participant criticizes the weight given to mildly divergent (not clinically relevant) lab results and its implications.

#### Theme 3: Treatment (in)possibilities

Pregabalin is the first choice for all but one, followed by tricyclic antidepressants (TCA) or duloxetine (parallel made with diabetic polyneuropathy) with no apparent preference. Nortriptyline is generally preferred over amitriptyline because of its side effects profile. In case of a circumscribed area of pain, capsaicin is used. Gabapentin, cannabinoids, other topical drugs, and opioids are incidentally prescribed.

Essential and individual patient education (on pain), coaching, self-sustainability, and recommending physical activity are sometimes reinforced by rehabilitation (daily problems and suffering), physical, occupational, and/or psychological therapy (anxiety and depression symptoms) (quote 68). This concerns existential issues, managing chronic disease and cancer, acceptance, and “creating a positive vicious circle” (quote 69). However, there is a shared frustration in seeing patients, having (had) cancer treated, now being disabled consequently, without sufficient treatment results.

By not thinking: ‘everything is always so painful’, but being active, keeping up activities, finding distraction and creating something of a positive vicious circle. Trying to improve energy levels, experiencing success, alleviating the experienced burden. … How do you cope with pain, which probably will not fade away totally, an aspect you should give attention to, improving acceptance, and probably giving space for improvements with our treatments (quote 69).

#### Theme 4: Socio-environmental aspects

Social and work-related issues are mentioned as a reason for further diagnostic efforts, although not yielding more therapeutic options (quote 70).

Most statements overlap with “theme 3 – treatment (im)possibilities.” The strategy in starting and increasing medication, coaching, creating options for self-sustainability, and weighing effects versus side effects are stated to be key in treatment success and patient satisfaction.

#### Theme 5: Diagnostic conundrums

In patients with pain, anamnesis (regarding any temporal relationship with chemotherapy cycles, distribution, comorbidities) combined with (specific) physical examination is regarded as sufficient by most. EMG is used in specific cases, regarded of little value in diagnosis and even less in predicting CIPN evolution. Questionnaires are not used. Interviewees are generally reluctant towards statements on the course of CIPN, especially in less used or newer chemotherapeutic agents, underlining the uncertain course and final state. (See also above “Theme 3.”).

#### Theme 6: Towards a proven standardized diagnosis and treatment

Scarce evidence on CIPN treatments results in preferences translated from the use in other neuropathic pain states. Three participants are researchers on anti-neuropathic drugs (partially in CIPN) or CIPN treatment specifically, recognizing trivial improvement in preventative, diagnostic, and therapeutic possibilities over the years. As mentioned, no specific local protocols exist resulting in variable CIPN care without notion of the scope of the problem, but blood tests are done according to the national neurologists’ guidelines. Participants are (all but one) unfamiliar with ASCO and/or ESMO CIPN guidelines, and thus do not apply them. Also here, some grip or framework for referral, diagnosis, and treatment is welcomed. Two participants participate in the development of national guidelines on (general) neuropathic pain.

### Pain specialists

In total, 63 descriptive codes were extracted from the data after coding, divided into ten categories and subdivided into five themes. A schematic overview of codes, categories, and themes is provided in Table 5.

**Table 5:** Pain specialists—codes, categories, and themes extracted from the interview data after coding

Codes	Categories	Themes
Treatment algorithm Anti-epileptic drugs Physical exercise Combination therapy Medication Treatment—miscellaneous Topical treatment Cannabinoids Capsaicin Ketamine Neuromodulation Treatment—non-drug treatments Opioids SNRIs Systemic intravenous therapy TCAs TENS Topical antineuropathic or analgetic drugs	Treatment regimen	Treatment modalities
Managing expectations Terminating treatment Treatment—guidance Prior to treatment Follow-up	Treatment path for pain	Treatment-related issues
Acute complaints Oncological treatment strategy Preventative measures Screening Chemotherapy type Patient education	Oncological treatment path	
Awareness Awareness—oncologists Treatment—side effects Treatment—effectivity Treatment—evidence Delay Case load versus prevalence	Restrictions	
Future treatments Evidence and research	Future	
Diagnosis—differential Diagnosis—specific tests Diagnosing CIPN characteristics Symptoms Clinical diagnosis Negative symptoms	Diagnosis	Diagnosis
Multidisciplinary consultations Cooperation—general Cooperation—different specialties Cooperation—oncologists	Multi- and interdisciplinary issues	Healthcare organization
General practitioner Neurologist Palliative care team Psychologist	Healthcare providers	
Organisational issues Physician assistants and specialized nurses Referrer	Organizational issues	
Patient approach Case report Influence on daily life Patient-centered Shared decision making	Personalized care	Personalized care

#### Theme 1: Treatment regimen

Generic, mainly local, and non-binding protocols are used in guiding CIPN treatment (quotes 71–72), prompting custom-made decisions depending on medical background, specific symptoms, and preferences (quotes 73–74). On referral, TCAs or gabapentin has often been used. Duloxetine is prescribed regularly as a first-choice alternative, and some prescribe anti-epileptics (e.g., pregabalin, gabapentin), while others dispute its value (quotes 75–77). Opioids are mainly used in a palliative setting, with general reluctance and varying ideas on its efficacy (quote 78). Occasionally, ketamine, cannabinoids, and other anti-neuropathic drugs are used (quote 79).

All subjects use capsaicin 8% patches, while other topical treatments (capsaicin 0.025–0.075%, lidocaine, phenytoin, and amitriptyline) are deployed to minimize adverse effects (quote 80). Some interviewees propagate the use of transcutaneous electrical nerve stimulation (TENS), but variable effects are expected, with diffuse pain localization impeding its practical use (quote 81). Singularly mentioned and seldomly used were electroacupuncture, iontophoresis, virtual reality, and neuromodulation (quote 82). Often, a physiotherapist for movement-related aspects or podologist is consulted (quote 83).

#### Theme 2: Treatment-related issues

Pain specialists are not actively involved during chemotherapy, and few are consulted for general advice in a palliative care context (quote 84). Preventive measures and improved screening would be welcomed (quotes 85–87), expecting an underrepresentation, and secondary complaints are common in late referral (quotes 103–105). Oncological issues and eye on survival tend to overshadow negative consequences of chemotherapy. This prompts adequate patient education on CIPN characteristics (e.g., positive, and negative symptoms relating to treatment possibilities), its influence on QoL, the efficacy of treatment possibilities, and thus expectation management (quotes 88, 93–94, 115–116). According to the interviewees, patients are generally not surprised by CIPN complaints per se, but by its severity (quote 96). The oncologists’ CIPN awareness is deemed sufficient, but not for pain and its treatment specifically.

Well, there’s not enough awareness about pain in general, or about clinical pain management teams. Many people stay with their general practitioner, or are not referred to us… so no, I believe there’s insufficient awareness about pain and pain management in general. (quote 98).

Medical management is assisted by specialized nurses or physician assistants for specific interventions and guiding a stepped-care plan, in multiple low-threshold and often telephonic appointments (quotes 89–92). When stable, and in absence of treatment possibilities, patients are usually followed up by their general practitioner (quote 95). Evaluation of treatment is often done with pain intensity scores (quote 99). Most are not convinced of the superiority of any treatment over another because of huge varieties in effect, and limited comparative evidence (quote 100). New scientific evidence is welcomed, but usually regarded as disappointing (quotes 101–102), explaining why multidisciplinary approach (supplementary) care is valued higher (quote 106).

Neuroprotective interventions, new topical treatment options, and prevention by pre-emptive analgesia or other drugs are regarded valuable topics in future research (quotes 107–108).

While oncologists are generally regarded the primary healthcare provider for CIPN patients, and a case manager secures continuity, interdisciplinary aspects and specific knowledge within other specialties should be applied more appropriately (quotes 109–110).

#### Theme 3: Diagnosis

Reported symptoms and signs, in a temporal relation to chemotherapy treatment, are considered essential and usually sufficient for diagnosis without additional testing (quote 111). Neurologists are only involved in unusual and severe cases (quote 112). Questionnaires assessing the multidisciplinary aspects of pain are always used (quote 113).

On referral, all patients report typical peripheral neuropathy complaints, and some report secondary symptoms (quote 114).

#### Theme 4: Healthcare organization

Multidisciplinary consultations within pain clinics, though usually not CIPN specific and only occasionally involving oncologists and other professionals, are considered essential in CIPN care (quotes 117–118). Content with low-threshold impromptu consultation (e.g., by telephone) is good, but the level of cooperation between specialties is variable. Mutual visibility (e.g., involvement in each other’s meetings), case discussions, and crossing the boundaries of one’s super specialized knowledge, possibly improving CIPN awareness and care, is time-consuming and an organizational challenge (quotes 119–121). Case managers, and changing attitudes over the years, are factors pointed out to have already improved this, mainly in palliative care (quotes 122–123).

A clear cutoff point for referral cannot be defined and should be tailored. Involvement seems valuable in complex cases, or if topical treatment is indicated (quote 124). As stated, delays in referral are common (quote 125). For optimal personal care and continuity, the specialized nurses’ or case managers’ role should be improved (quote 126).

#### Theme 5: Personalized care

As stated, patient education and instructions, adequate management of expectations (also related to pre-existing functionality and activity level), and identifying confounding psychosocial factors (e.g., anxiety, coping strategies) are important (quotes 127–128), implying the importance of a multidimensional approach and shared decision making (quotes 129–131).

I think the right guidance and education is crucial, especially for neuropathic pain, because success rates are so low. So, you really have to… patient empowerment is really important. (quote 127).

Having regrets after chemotherapy does occur, up to one request for euthanasia in intractable CIPN, but is usually perceived in the light of uncertainty on forehand (quotes 132–134).

## Discussion

In this study, by interviewing multiple specialists with different expertise, we gain a broader insight in factors influencing cooperation, referral, diagnosis, and treatment. Concluding points of specific interest and common denominators, which form the basis for this discussion, are provided in Table 6.

**Table 6:** Conclusions per specialty

	Medical oncologists	Neurologists	Pain specialists
Standardizing care	Algorithms partially embedded in chemotherapy protocols	Use of protocols on peripheral neuropathy in general	Use of protocols and guidelines on other (neuropathic) pain states
	Existing ASCO and ESMO CIPN guidelines [11, 12] are seldomly applied in the absence of (local and national) protocols, despite the wish for some grip on CIPN care as a wide variation in care, referral, and the specialists’ role is noticed		
Multidisciplinary aspects	Usually starts treatment	Focus on functional aspects Specific value regarding (differential) diagnosis	Specific value regarding treatment options and multidisciplinary options
	Initiatives on multidisciplinary consultation and individual specialization depend mainly on local possibilities, cooperation between specialties (existing partnerships/ meetings), and patient flow		
Prevention	General—practical advice Pre-existing peripheral neuropathy leading to 25–50% chemotherapy dose reduction	Not (often) involved	Not (often) involved Focus on the prevention of secondary complaints by early referral
Informed consent	Focus information on symptoms, irreversibility, uncertainties, and the influence on oncological treatment (survival)	Focus on weighing effects versus side effects	Focus on treatment (im)possibilities The intensity of CIPN complaints overwhelms patients
Screening and diagnosis	Brief verbal screening CTCAE testing to indicate chemotherapy dose reduction PROMs only in research	Lab testing in accordance with national guidelines EMG in specific cases, deemed of little value	Focus on temporal relationship of complaints and chemotherapy General questionnaires used
	First choice: TCAs and anticonvulsants without specific preference Occasional specific support regarding practical aspects or coping	First choice: pregabaline and nor- or amitriptyline Focus on patient education (on pain), coaching, self-sustainability, and physical activity	First choice: duloxetine and TCAs or capsaicin 8% patch Involved in treatment resistant cases Focus on multidimensional aspects and the management of expectations
Treatment	Other than first-choice antineuropathic drugs, capsaicin 0.025–0.075%, ketamine, cannabinoids, and opioids are applied Choices and evidence are partially translated from other neuropathic pain states Lifestyle advice and paramedic expertise (physical therapy, podiatry, occupational therapy), psychological care (items: explanation, pain education, acceptance), and supportive care or rehabilitation therapy are universally deployed		
Follow-up	Differs per oncological setting and chemotherapy regimen	Variable views on the value of (frequent) visits	Implementing a stepped-care plan in frequent contacts, supported by specialized nurses or physician assistants
Areas of improvement	Awareness and attention Available (epidemiological) data Value of exposure to CIPN	Little improvements in possibilities recognized over the past decade(s)	Vital role seen for specialized nurses or case managers for optimalization of personal care and continuity
	Wish for effective strategies for treatment and prevention Standardization of CIPN care and the use/implementation of existing protocols		

Few published studies explore clinician and patient perspectives on the provision of care, information, and support. Their approach is mostly clinician- and oncology-centered, focusing on technical aspects and knowledge. In a study by Knoerl et al. [15], CIPN symptoms were discussed and documented in less than half of the encounters with patients at risk. There is evidence for the use of QLQ-CIPN20 and FACT/GOG-Ntx in a research setting, but data on the use of PROMs in a clinical setting is limited. While multiple studies found a lack of familiarity with CIPN in patients as well as clinicians, a study on the use of a web-based care planning system did not directly underline its necessity in identifying symptoms [15–18]. Also, CIPN diagnosis is not straightforward, no single method is advised over another in ESMO- and ASCO guidelines, and important regional differences in assessment, incidence, and prevalence occur [19].

Despite the wish for a “who-what-when-structure” in diagnostic (specific tests), treatment (evidence, regimen), and organizational (referring, follow-up) aspects, few local or national protocols are available and international guidelines are scarcely used. Delayed treatment or referral is encountered and most welcome early referral and interdisciplinary consultation, while some question its (cost-)effectiveness. To our opinion, better acquaintance with international guidelines could provide an obvious first step. In this context, attention for implementation and evaluation also seems important when publishing new guidelines. Secondly, focusing on organizational aspects, and national and regional protocols could be developed. Thirdly, better registration of complications including CIPN as suggested by multiple specialists could help identifying patients at risk, improve awareness, and identify shortcomings in current policy. A recent Delphi study among seven respondents on 18 statements within four themes (pre-treatment review, screening and assessment, management and referral, and CIPN path feasibility) regarding a new-developed clinical pathway recognized its value in CIPN care across different health services [20].

Diagnostic strategies vary, lacking a standardized approach. A fitting complaint pattern in temporal relation to chemotherapy suffices for most. Ideas on the use of rating scores (mainly in research and by oncologists) and more invasive tests (blood tests for co-morbidities and EMG, mainly by neurologists) were proposed. However, PROMs are not used in diagnosis. While their value is proven for some diseases and pain conditions, the clinical value in CIPN is less established [18, 21, 22].

The lack of treatment possibilities in painful CIPN is generally deplored, which influences clinical practice, for instance regarding anti-neuropathic drugs (no high hopes, NNT perceived as high) or referral (“what’s the use”?). Again, a structured approach is wished for. Treatment varies and is rated as “random,” influenced by personal experience or translation from evidence for other pain states, but usually consists of chemotherapy dose reduction or cessation (oncologists) and anti-neuropathic drugs (all specialties), while other (multidimensional) options are occasionally used. Acquaintance with topical treatment could possibly be improved, and the copying of a therapeutic approach/regimen from other neuropathic pain states and/or polyneuropathies raises the question whether this is adequate and cost-effective. For instance, only duloxetine is recommended in ASCO guidelines on CIPN, (partially) explaining therapy failure as described by several professionals (for instance in medical oncologists; thema 3: therapy, quotes 29–36). The consequences of changing the chemotherapy regimen can be profound. These matters fit patients’ wishes: a need for a focus on prevention instead of treatment, and a greater focus on non-pharmacological treatments. Also, the possible value of exercise in CIPN prevention is recognized yet under-utilized, whereas in ESMO and ASCO guidelines, supervised medical exercise as well as self-management interventions for treatment and even prevention are advised [11, 12, 21].

Opted ideas for improvement can be seen as starting points for future protocols, guidelines, and research [21]. Most feel a need for standardization in diagnostic, screening, and treatment approaches. A general need for better treatment options (medication or otherwise) is felt, but in general expectations are low (all specialties). Follow-up seems to be of a very “technical” nature, symptom driven, with little focus on QoL. The possibilities and value of a more multidisciplinary approach (e.g., psychological, multidimensional/rehabilitation, general guidance) should be further explored, as many specialists claim its value from the perspective of chronic pain and suffering (this study, 19).

Our study has some limitations. First, bias in selection of the interviewees is conceivable, due to recruitment within affiliated hospitals and professional network and an above average interest in the subject. Second, other specialists and the general practitioners participate in CIPN care. Their exposure however is probably limited, co-incidental, and variable, so that interviewing them might not yield representative results. Third, patients’ perspective and optimizing shared decision making other than merely providing information would be of interest [13].

In summary, interviewing different specialists generates a broad insight in the clinical approach of CIPN and differences between specialties. Key outcomes are displayed in Table 6, but foremost a structured approach towards diagnosis, treatment, referral, and follow-up is welcomed. This could partially be accomplished by improving knowledge and the application of existing (international) guidelines. For a clearer “who-what-when,” national and regional guidelines on CIPN specifically, instead of using those on other (neuropathic) pain states, could be developed. Interdisciplinary consultation and meetings are mentioned, but their value for all or specific CIPN patients is to be determined. In further research, besides better therapeutic options for CIPN, the patient perspectives, needs, and wishes should be explored.

### Supplementary Information

Below is the link to the electronic supplementary material.

Supplementary file1 (PDF 154 KB)

Supplementary file2 (PDF 543 KB)

## Data Availability

No datasets were generated or analysed during the current study.

## Declarations

This study was performed in line with the principles of the Declaration of Helsinki. This explorative qualitative study with semi-structured interviews was performed after approval by the local research ethical committee (METC AmsterdamUMC (VU), Amsterdam, The Netherlands, dossier number: 2021.0069). (See also the “ Methods ” section of the manuscript.)

Registered oncologists, neurologists, and pain specialists, involved in the care for patients with CIPN, were invited to participate in this interview study. Written (e-mail) and oral information was provided (practical, on anonymity, on withdrawal) and informed consent for participation and publication was obtained before any actual interview. (See also the “ Methods ” section of the manuscript.)
