# Supplementary material for: A qualitative evaluation of the oncologists’, neurologists’, and pain specialists’ views on the management and care of chemotherapy-induced peripheral neuropathy in The Netherlands
Source: Support Care Cancer. 2024 Apr 22;32(5):301. doi: 10.1007/s00520-024-08493-4 (PMC11035431; doi:10.1007/s00520-024-08493-4)
Supplement: Supplementary file 1 — Supplementary file1 (PDF 154 KB) [file 520_2024_8493_MOESM1_ESM.pdf]

Medical oncologists:

1.1

Quote 1 - “There have been a lot of studies that added things like magnesium, and I don’t know what else, but all these studies turned out to be inconclusive, so we don’t do that anymore” [participant 5]

Quote 2 - “I don’t think that there are any serious effective therapies in preventing CIPN. There are some studies performed, but these studies didn’t yield any effective treatments.” [participant 6]

Quote 3 - “And with regard to alcohol consumption, we always advice patients to stop drinking alcohol during chemotherapeutical treatment due to the central neurotoxicity it can endure. At the beginning of the treatment, we tell patients that they can’t drink any alcohol in the first week.” [participant 3]

Quote 4 - “We don’t take risk factors that much into consideration in developing a treatment plan. (…) I don’t think that it plays that big of a role in the development of CIPN.” [participant 5]

Quote 5 - “Well, besides diabetic neuropathy and alcoholic neuropathy I don’t use any other risk factors.” [participant 4]

Quote 6 - “I used to think that patients with diabetes were more vulnerable to develop CIPN. However, when studies revealed that the link between diabetes and the risks of developing CIPN weren’t that strong I became less cautious when treating these patients.” [participant 3]

Quote 7 - “Well, if the patients don’t have any form of neuropathy already, it (risk factors) hardly plays any role. Actually, no they don’t play any role.” [participant 6]

Quote 8 - “We do often refer the patients back to their own diabetic nurse practitioner to ensure that the glucose levels are strictly regulated during the course of those periods.” [participant 8]

Quote 9 - “I think that we see it (CIPN) quite often, and the problem resolves itself most of the time (…) What you really need is something that enables you to target those patients that will develop long-term symptoms.” [participant 3]

Quote 10 - “In some cases, you stop the treatment and ask yourself: ‘did I stop to early?’ and in other cases when a patient develops symptoms you think: ‘why didn’t I stop earlier?’.” [participant 7]

1.2

Quote 11 - “I always inform the patient verbally during the conversation, but I also give the patients a written handout in which the treatment is explained. CIPN is amongst one of the items that is addressed in that document. I always inform the patient that oxaliplatin has two

forms of neuropathy, being acute neuropathy that is triggered by cold temperatures. And that there is a chronic cumulative form of neuropathy that can lead to more sensory symptoms." [participant 8]

Quote 12 - "I tell my patients that oxaliplatin can do a few certain things. (…) That the symptoms can get worse if you use more oxaliplatin. And that the symptoms don't always disappear if you stop the treatment, they can get even worse. The symptoms can be reversible, but this sadly isn't always the case." [participant 6]

Quote 13 - "Most patients are more eager to continue with the chemotherapy because they want to attack the cancer with all we've got. But it is important to council them on the effects this can have on the course of their symptoms, and that it can result in chronic or worse symptoms. These symptoms can be really invalidating and if you reduce the dose of the chemo it can work just fine." [participant 4]

2.1

Quote 14 - "The screening on peripheral neuropathy is performed during the anamnesis. And you are extra careful treating patients that already are at risk of developing peripheral neuropathy due to for example diabetes. (…) And sometimes you refer a patient to a neurologist to get a better understanding of what causes their symptoms." [participant 4]

Quote 15 - "What I always tell my patients is that they have to promptly alert me whenever they feel symptoms of CIPN. I explain that it is a nasty side-effect, and that I can't always solve the problem. And that that is exactly why they have to alert me, because the damage does not clear out when you stop with your chemotherapeutical treatment." [participant 9]

Quote 16 - "In the end, the most important part of the treatment is that toxicity is assessed before every round of chemotherapy by a physician, nurse practitioner or floor nurse." [participant 3]

Quote 17 - "I mainly use the CTCAE grade, you surely know about this scale, do you? It is a pretty broad scaling method but well… it is widely adapted in clinical practice and most of the times I use it as well." [participant 7]

Quote 18 - "The tool we use to grade CIPN is the CTC toxicity criteria. We use that to score the symptoms of CIPN in patients." [participant 9]

Quote 19 - "In normal circumstances we do not use questionnaires. However, if the patient participates in certain studies some more elaborate questionnaires are used, but not in normal clinical practice." [participant 7]

Quote 20 - "In normal clinical practice, we only use the anamnesis. Although nearly all patients do participate in some form of questionnaire-based research which also contains a questionnaire regarding neuropathy." [participant 6]

Quote 21 - “Whenever I give patients an adjuvant treatment, in this hospital the patient visits the surgeon as part of their follow-up. I do not see the patient myself anymore. However, I do see patients if they have a bad case of CIPN and these patients also know that they can always contact me if they need me.” [participant 2]
Quote 22 - “Normally the follow-up after three months is performed by the surgeon. But if there is any form of neuropathy the nurse practitioner will keep in touch. As a sort of aftercare.” [participant 3]
Quote 23 - “That is a good question, if the malignancy has metastasized the contact never stops. Or well… only if the end is near.” [participant 6]

2.2
Quote 24 - “I think we diagnose them at the outpatient clinic. Either we or the nurse practitioner. If the symptoms fit the description, and the patient is treated with chemotherapy we make the diagnosis and grade them according to the CTC toxicity criteria.” [participant 9]
Quote 25 - “No I only use the anamnesis; the neurologist performs additional tests. I don’t think that I am skilled enough to perform and interpret those tests like a neurologist.” [participant 4]
Quote 26 - “Well if there is an uncertainty or when patients can’t describe their symptoms clearly, I tend to ask a neurologist to advise me.” [participant 5]
Quote 27 - “Yeah, sometimes patients experience other symptoms besides the typical sensory loss and cold allodynia such as a foot drop. So, in that case, when a patient experiences both motor and sensory symptoms, can be a reason for me to consulate a neurologist. (…) If you just can’t explain the symptoms as being CIPN I do refer my patients to a neurologist.” [participant 4]
Quote 28 - “The moment a patient presents him/ herself with complaints of pins and needles in the right foot and doesn’t have any symptoms in his right foot I tend to think that something might be off. Are we dealing with CIPN or are these symptoms caused by something else?” [participant 6]

3.1
Quote 29 - “And I suggest some pharmacological options to treat the neuropathic pain such as pregabalin, amitriptyline or nortriptyline.” [participant 2]
Quote 30 - “Some patients tell me that they can’t sleep due to the pain. If patients can’t sleep, I frequently start a form of medication such as amitriptyline before the patient goes to sleep.” [participant 3]
Quote 31 - In response to: “Is duloxetine ever used in treating painful CIPN?”

“Yes, but I don’t prescribe that personally. Only the pain specialist prescribes duloxetine in our hospital.” [participant 8]
Quote 32 - “I also advice my patients to wear firm shoes, don’t use decorated socks with a lot of seams, inspect your feet. More practical advice.” [participant 9]
Quote 33 - “I always advice my patients to avoid cold objects and drinks. If you have to get something from the freezer, let someone else do that for you. And wear warm socks and cloves in cold weather!” [participant 5]
Quote 34 - “A patient of mine had a real bad case of painful CIPN. I started neuropathic painkillers the patient stopped after a while because he was experiencing to many side-effects. And the effect of the therapy was quite disappointing to be honest.” [participant 2]
Quote 35 - “Depending on the symptoms that a patient is experiencing I try to find the best supportive care for a patient like a physiotherapist or an occupational therapist.” [participant 6]
Quote 36 - “If a patient is suffering from numbness and the chemotherapy treatment is already finished, I advise them to take good care of their feet, and sometimes I refer them to a podiatrist.” [participant 8]

3.2
Quote 37 - “If a patient already has a bad case of peripheral neuropathy this will mean that I won’t start oxaliplatin. And if the patient has a mild form of peripheral neuropathy I will start in a reduced dose. For that I use the CTC criteria, grades 1 and 2 get a reduced dose, grade 3 doesn’t get oxaliplatin.” [participant 5]
Quote 38 “And that is why I always ask what a patient’s occupation is. If the patient highly depends on his or her fine motor skills such as a professional guitar player, the smallest amount of peripheral neuropathy can lead to tremendous problems.” [participant 6]
Quote 39 - “Every day we have a meeting with all oncologists and other specialists where we discuss all new patients in the outpatient clinic. We help each other decide on what course is best such as starting with a reduced dose or starting with a normal dose but reducing it as soon as mild symptoms come to light.” [participant 3]
“Yes, we do reduce the dose of the oxaliplatin when we encounter neurotoxic side-effects. We first reduce the dose to 75% and then if the patient continues to have grade 2 symptoms, we reduce to 50% but don’t go further. This is all noted in a sort of roadmap that guides us called ‘the mother protocol’.” [participant 1]
Quote 41 - “It is a risk assessment with each type of treatment. If you treat a patient in a palliative way with liver metastasizes and the patient can tolerate the symptoms, you want to go for the maximal response to improve the survival. Whereas patients that are treated with adjuvant chemotherapy I am more likely to reduce the dose of the chemotherapy because

they already had a curative surgery and are therefore at a lower risk. It is an assessment that I have to make, and sometimes I refer to my collogues to help me." [participant 3]
Quote 42 - "In that way if you treat a patient in a palliative way, it differs from when you treat a patient with adjuvant chemotherapy because in a palliative setting you tend to accept more toxicity." [participant 9]
Quote 43 - "Everything you decide is done through shared decision making. And when you reduce or stop the oxaliplatin you inform the patient on how they might expect the symptoms to develop over time." [participant 6]
Quote 44 - "Sometimes we do start with oxaliplatin in patients with pre-existing neuropathy, after consulting a neurologist of course, because that patient had a different type of neuropathy" [participant 4]
Quote 45 - "If I don't treat a patient then they might have a low chance of getting CIPN, but they have a 100% chance of dying. So it is really important that you discuss with your patient what side-effects he or she is willing to accept." [participant 9]

4.1
Quote 46 - "Well to be completely honest, I don't know. Maybe it exists, but just like many other of these protocols, if they exist, they exist, but we don't use them very often to be honest." [participant 2]
Quote 47 - "In every treatment schedule it advises you on what to do at a certain level of neurotoxicity. In the case of oxaliplatin, which is the most feared of all chemotherapy's regarding neurotoxicity, it advises you very clearly on how to react. Meaning stopping or reducing the dose of oxaliplatin." [participant 6]
Quote 48 - "So it is not a specific peripheral neuropathy guideline, there are treatment guidelines that state CIPN as a side-effect and it advises the physician on what they should do." [participant 6]
Quote 49 - "And these so-called 'care-programs' provide an overview of what chemotherapy you use for each type of malignancy, and it also suggests alternative treatment options if the patient is already experiencing symptoms of peripheral neuropathy." [participant 3]
Quote 50 - "We mainly use the patients' files to note down that a patient has CIPN. We do not have some sort of database or something like that." [participant 8]
Quote 51 - "So maybe it's an idea to gather information of all CIPN patients in the Netherlands because I have the feeling that it doesn't occur that often. This might enable researchers to perform better studies that maybe formulate new insights to improve the care of these patients." [participant 3]

Quote 52 - “I always write down in my letter to the general practitioner that certain symptoms can occur due to the chemotherapy. And if these symptoms do occur, they can always contact us for advice.” [participant 8]
Quote 53 - “I refer my patients to a neurologist if I’m not sure if CIPN is causing the symptoms. I refer my patient to the pain specialist if I can’t treat the pain caused by CIPN on my own with gabapentin, pregabalin or amitriptyline.” [participant 6]
Quote 54 - “I would refer my patient to a neuro-oncologist rather than referring them to a neurologist or pain specialist. We have a team of neuro-oncologists that have specialized themselves in CIPN so I always refer my patients to them.” [participant 1]
Quote 55 - “Depending on the symptoms that a patient is experiencing I try to find the best supportive care for a patient like a physiotherapist or an occupational therapist.” [participant 6]
Quote 56 - “If a patient is suffering from numbness and the chemotherapy treatment is already finished, I advise them to take good care of their feet, and sometimes I refer them to a podiatrist.” [participant 8]

5.1
Quote 57 - “I think that CIPN deserves more awareness, but that is the reason why you are doing this. Maybe more research is a good start? Research is something that can always unites people.” [participant 2]
Quote 58 - “I think that it can be a good start to gather more information of our own patients so that we can perform more epidemiological studies on Dutch patients. With that we can also create some more awareness I hope.” [participant 3]
Quote 59 - “There is a lack of scientific evidence about CIPN, and as far as I know there is no one that draws the attention to it. It is sort of the orphan of the symptom oriented oncological healthcare. (…) It would be nice if we could create some more awareness about CIPN and make physicians more attentive to diagnosing and treating it.” [participant 5]
Quote 60 - “You have to try to prevent it by reducing the dose of oxaliplatin in a timely manner. I know that because I use it quite frequently. However, not all physicians use oxaliplatin that much and aren’t that strict in assessing their patients on the early signs of CIPN. And they tend to be overwhelmed by thy symptoms if they suddenly occur.” [participant 4]
Quote 61 - “And I also think that physicians that don’t use oxaliplatin that often, I mean I is not part of every oncological treatment regiment, that they might not be that attentive towards CIPN which can lead to some missed or delayed diagnoses.” [participant 6]
Quote 62 - “If you could standardize the diagnosis and treatment or provide guidance, I think that would be of great help for everyone.” [participant 2]

Quote 63 - “If there were any other options than reducing or stopping the treatment that would be great. I myself am right now working on a study to see if there are any other effective treatment regiments without oxaliplatin that are just as effective. Or if we had something that reduces the neurotoxicity of oxaliplatin. I mean, oxaliplatin is a really effective drug, it really is. So, if you can reduce the side-effects that would be really welcome. Yeah, I think there is a real clinical need for this.” [participant 6]

Neurologists:

1.1
Quote 64 - “I think variation is large. This does not necessarily have to be wrong; some doctors feel more comfortable starting specific treatments than others. But some framework, some agreement… who will start treatment, which patient needs a neurologist or a pain physician… Yet then, what exactly ís the best treatment? To my opinion, even that is not certain. So something practical: who, what, when? That, I would welcome.” [participant 4]

1.2
Quote 65 - “Of course, there are patients telling a crystal clear story, saying: ‘well, I have numbness and pain in both feet, and when I get out of bed I have to turn on the lights not to tumble over’, with an exact time line, and with some simple neurological physical examination… but others do not!” [participant 4]
Quote 66 - “Because I can imagine that oncologists do not refer patients to us, claiming we don’t add much. Which, unfortunately, for some patients, is true.” [participant 1]

1.3
Quote 67 - “If there is no pain, I just explain the nature and usual course of their problem. With practical and mobility related issues a physical therapist could add value in for instance stable standing and walking. But if there is pain, I start treatment, and follow-up patients.” [participant 4]

3.3
Quote 68 - “That depends. In extreme pain, rehabilitation could be a good step, focused on pain. This would be if there is a significant problem, when pain is always in the foreground, when a patient doesn’t leave the house so to say. But if it is a specific problem of balance, or loss of sensation, or their shoes are never comfortable, …, then low-frequency rehabilitation, more general, would suffice.” [participant 4]

Quote 69 - “By not thinking: ‘everything is always so painful’, but being active, keeping up activities, finding distraction and creating something of a positive vicious circle. Trying to improve energy levels, experiencing success, alleviating the experienced burden. … How do you cope with pain, which probably will not fade away totally, an aspect you should give attention to, improving acceptance, and probably giving space for improvements with our treatments.” [participant 2]

4.1
Quote 70 - “*In young people, I am tending to do more tests or refer them. So, in the context of health insurance, and as a declaration towards their employer, they can ‘proof’ their complaints.”* [participant 2]

Pain physicians:

1.1
Quote 71 - “… we’re working on care pathways, to show others when referrals could be purposeful or not, and we would like to do the same with peripheral neuropathy. But, mainly to point out where a real difference can be made… or to indicate when a possible referral won’t matter.” [participant 8]
Quote 72 - “But it’s complicated, because these patients, they… they blend with other patients with peripheral neuropathies… so that’s a mixed group. So, to include them all in one protocol, that’s quite difficult” [participant 3]
Quote 73 - “If someone really experiences allodynia or pain from light touching, then I’d rather go with [topical] lidocaine and treatment with capsaicin.” [participant 1]
Quote 74 - “There are people who experience partial relief of symptoms, and they might say: “I do notice some difference, but it’s not acceptable yet”. That might be a reason to combine Qutenza [capsaicin patches] with anti-neuropathic medicines” [participant 7]

1.2
Quote 75 - “Look, amitriptyline… well all the TCAs actually, they’re all a bit like a scattergun, it does something on all the receptors. The problem is people experience more side-effects… I think that’s the advantage of duloxetine, it gives a little less adverse effects, or less invalidating adverse effects, than amitriptyline.” [participant 3]
Quote 76 - “For example, I take into account the fact that amitriptyline causes drowsiness, so generally when patients with neuropathy experience worsened sleep at night, that would be my first choice. But because of the lack of evidence concerning CIPN, I’m not really

inclined… it’s not in my top 3 choices, because at the very least, there is some evidence for CIPN [regarding those three].” [participant 5]
Quote 77 - “I don’t think I have ever seen a patient who experienced any relief from Lyrica or pregabalin, to be honest. With this disease, no.” [participant 5]
Quote 78 - “If you’ve already tried anti-neuropathic treatment that doesn’t work, or there are too many side-effects, opioids are absolutely allowed and they definitely work. But it’s medication you have to keep your eye on. … I think there’s a NNT of 3.5 for antidepressants for neuropathic pain, and I believe it’s like 4.2 or something for opioids, that’s better than gabapentinoids. So opioids definitely work, and effectively, and more efficient than most anti-neuropathic treatment. That’s something that shouldn’t be forgotten, especially for patients dealing with oncological disease.” [participant 9]
Quote 79 - “If duloxetine isn’t working, what is venlafaxine going to do?” [participant 8]

1.3
Quote 80 - “The nervous system is also your brains, so everything affecting the nervous system, also affects your consciousness… that’s the problem with this disease.” [participant 2]

1.4
Quote 81 - “It’s very difficult, because symptoms are diffuse. Mostly, there’s not just one area, but… both feet, so left and right. So that’s a real inconvenience when using TENS for CIPN.” [participant 6]

1.5
Quote 82 - “We work with two pain psychologists regularly, and they use… well don’t ask me what it’s called exactly, but they do use techniques… I tell my patients they use techniques to reset their brains. So basically, what they do is, they use psychological interventions to try and change the experience of pain of the patients. So, they can try and ignore the pain, to push it further to the background.” [participant 4]

1.6
Quote 83 - “Altered footwear is something almost no patient has, they often don’t even know it exists. Most patients have never heard of a podologist.” [participant 9]

2.1
Quote 84 - “I want them earlier in the process, but do I want them during chemotherapy? I’m not sure, my interfering might worsen their [complaints].” [participant 5]

Quote 85 - “Pain can also be… for patients with oncological disease, it can also be an indicator.” [participant 8]
Quote 86 - “Well, first you have a look at already prescribed and tried medication, so if someone already tried combining two or three substances, like pregabalin, amitriptyline, you name it… nortriptyline, obviously all the well-known drugs… ” [participant 2]
Quote 87 - “I believe that a specialized nurse at the oncology department who specifically pays attention to CIPN complaints, that might result in several patients not having to visit a pain specialist. I think it’s important to start some kind of therapy early on.” [participant 2]
Quote 88 - “Well, I also work in Bonaire, and over there it’s sort of standard for patients to record the conversation, because they don’t understand a lot of things. So then afterwards, they can just listen to it again.” [participant 1]

2.2
Quote 89 - “So, I call the patients, or I ask the physician assistant to do that for me, to inventory possible side-effects of the medication.” [participant 1]
Quote 90 - “When patients get the Qutenza treatment, the first checkup we execute ourselves, later on that’s the responsibility of the specialized nurse, the pain nurse.” [participant 4]
Quote 91 - “Right now, we perform the follow-up ourselves, we did divide it between the pain specialists and nurse practitioners in the past, but… yeah it caused a bit of a shredded entity.” [participant 8]
Quote 92 - “Well right now the follow-up is dramatic, in this corona-time, because you’re not allowed to invite your patients to visit the hospital unless it’s absolutely necessary.” [participant 1]
Quote 93 - “First of all, I start with explaining what CIPN is, then secondly, I immediate try to mitigate expectations. Explanation is based on negative and positive symptoms, obviously, and the fact that neuropathy is very difficult to treat, especially when it’s caused by chemotherapy.” [participant 5]
Quote 94 - “According to me, there are no other treatment strategies for these patients. And I’m particularly honest about this fact to my patients.” [participant 4]
Quote 95 - “The less doctors, the better” [participant 5]

2.3
Quote 96 - “What is it like to not be able to button your own shirt, do you think you’d be able to envision that?” [participant 5]
Quote 97 - “Regarding awareness, we have some old-fashioned oncologists… they do not pay a lot of attention to this.” [participant 4]

Quote 98 - “Well, there’s not enough awareness about pain in general, or about clinical pain management teams. Lots of people stay with their general practitioner, or aren’t referred to us… so no, I believe there’s insufficient awareness about pain and pain management in general.” [participant 1]

Quote 99 – [When asked which intervention showed greatest result in clinical practice] “I’m going to be honest with you, I don’t think I can give you a clear answer…?” [participant 6]

Quote 100 - “I know the existing evidence for CIPN recommends duloxetine, but in daily practice… I cannot confirm that. In my experience over the last decades, it doesn’t really matter which antidepressant you choose. Personally, I pick the one most suitable for my patient, their characteristics and wishes.” [participant 9]

Quote 101 - “If you’re trying to help people with really intense chronic pain, yeah well, that’s a reason to use the entire bag of tricks, unfortunately without a lot of success.” [participant 5]

Quote 102 - “Yes of course that would be nice, very nice indeed!” [participant 4]

Quote 103 - “When patients get to my consultation hour for the first time, they mostly have been experiencing symptoms since at least half a year until… well, yeah, much longer, years even. So naturally, they already have secondary effects and complaints, with their hair, nails, the arches of their feet.” [participant 9]

Quote 104 - “I think CIPN occurs a lot, far more than we get to see, I think.” [participant 3]

On the contrary, the same subject warns for probable overtreatment, as CIPN is believed to be partially self-limiting.

Quote 105 - “… and I think CIPN is undertreated right now. On the other hand, we should watch out for overtreatment, as we know that a part is self-limiting, but I don’t think there’s a lot of clarity about this phenomenon.” [participant 3]

2.4

Quote 106 - “Right now, I don’t believe there is any other treatment strategy suitable.” [participant 4]

Quote 107 - “I think we should… a lot of research is actualized by specialists working at academic medical centers, but it’s also very important to engage other, regular hospitals. I mean, how many doctors work in academic centers? Most of them work in regular hospitals.” [participant 1]

Quote 108 “So, maybe we could try starting with anti-neuropathic analgesia before chemotherapy? I’m not sure, but maybe that would be something to look into, to do some research on.” [participant 6]

Quote 109 - “… I think we, as pain specialists, could be mostly advisors, but the oncologists should stay the lead.” [participant 9]

Quote 110 - “We would like specialized nurses to act like the hinges between intramural and extramural care, a bit like a case manager, that’s a role these specialized nurses could fulfill.” [participant 3]

3.1

Quote 111 - “So we look for clinical signs with cotton buds, pin pricks, cold touch, that sort of thing. But we don’t execute a wide variety of other tests, no. We also don’t have time to do so.” [participant 1]

Quote 112 - “Well, if you would involve a neurologist in the diagnostic process, he or she will obviously find the same deviations based upon the EMG.” [participant 6]

Quote 113 - “For example, we use the HADS [Hospital Anxiety and Depression Scale, red.] when we suspect a patient to have an additional affective problem.” [participant 6]

3.2

Quote 114 - “Mostly they experience the classical neuropathy symptoms, but with a severity… with great severity of these complaints, struggling with hefty restrictions concerning physical and psychological areas.” [participant 9]

Quote 115 - “Sometimes, it’s not clear if patients really struggle with painful symptoms, or that they mainly experience negative symptoms. There tends to be confusion about this, so sometimes we also get patients with negative symptoms referred, but we can’t help them… so we have to disappoint those patients.” [participant 5]

Quote 116 - “… and if those [negative] symptoms are treated sufficiently, for example using physical or occupational therapy, that causes neuroplastic changes, resulting in a decrease of pain symptoms. ” [participant 9]

4.1

Quote 117 - “I think there aren’t a lot of available treatment options, so eventually, we’d all end up with the same conclusion. So I don’t believe discussing the options in a multidisciplinary consultation adds a lot of value. ” [participant 4]

Quote 118 - “Well, I think there’s not enough in the existing guidelines about supplementary care and interventions. … So, in my opinion, a primarily multidisciplinary approach is absolutely worthwhile.” [participant 9]

Quote 119 - “Maybe we should determine how many patients we missed out now that we don’t cooperate a lot with the oncologists. If we would be more involved in that multidisciplinary consultation moment. But because we don’t assess or quantify that number, we don’t really know, right?” [participant 6]

Quote 120 - “No, the pain specialists are not involved in the consultation moments of the oncology department. And we also don’t have enough time to do so.” [participant 5]
Quote 121 - “I think recognizing each other’s possibilities, as well as limitations, that’s always a good thing. I believe there’s a lot we don’t know about each other, so a lot of doctors have no idea what their direct colleagues or from other medical specialties, are doing in their daily practice.” [participant 2]
Quote 122 - “I think our oncologists who are a bit older aren’t as receptive for the involvement of a pain management team. And that’s a good thing, in the early stages, but… I think they don’t appreciate the added value a pain management team can provide. So very slowly, we try to change that in our hospital, but it takes time. And then the new, younger colleagues, they’re educated differently, they have more attention and appreciation for these things.” [participant 4]

4.2
Quote 123 - “… we even have a fourth health care provider, another doctor’s assistant, involved in the Qutenza treatment, if you’re counting all people involved.” [participant 4]

4.3
Quote 124 - “After completing curative oncological treatment, dialogues between oncologist and patient are very different than before. When treated successfully, these conversations are about the fact the cancer didn’t return, and how grateful the patient is. So he or she is not going to complain to the oncologist about the pain in hands and feet. So I believe that’s the moment to refer a patient to a pain specialist, that would be the turning point.” [participant 5]
Quote 125 - “The outpatient clinic is always packed with people, I sometimes say the number of patients is unlimited.” [participant 8]
Quote 126 - “A lot of oncology departments also have some kind of… it’s not a buddy, but someone who sort of guides the patients along their journey through the hospital… oh yeah, a case manager!” [participant 9]

5.0
Quote 127 - “I think the right guidance and education is crucial, especially for neuropathic pain, because success rates are so low. So you really have to… patient empowerment is really important.” [participant 5]
Quote 128 - “If a patient used to be very active, CIPN is much more invalidating than for patients who weren’t doing a lot anyway. On the other hand, someone who is very busy, might accomplish to deflect their attention to those activities. So it really depends on the

severity of the symptoms and the location, hand or feet, and the things someone was used to doing before chemotherapy and the onset of CIPN.” [participant 2]

Quote 129 - “When someone finally gets referred to a pain specialist, it’s mostly about adding supplementary care and treatment, so multidisciplinary interventions. So that patients learn to live with their disabilities.” [participant 9]

Quote 130 - “If you’re cured of your cancer, that’s different, than hopefully you can learn to accept that CIPN is a consequence of the chemotherapy that contributed to eradicating the cancer. But if you only have 5 more years to live, of which you’re dealing with CIPN for 3 years… that’s really, really troublesome.” [participant 2]

Quote 131 - “The question remains: is it ever a real deliberation, to undergo or not undergo chemotherapeutic treatment? I think very often, there’s not really a choice to be made… they just go on with it.” [participant 7]

Quote 132 - “Most patients say: ‘I’m still happy to have gone through with chemo, but this is also really bad’. One individual actually said: ‘If I’d known this in advance, I wouldn’t have gone through with it’.” [participant 8]

Quote 133 - “Obviously, in hindsight it’s very easy to say: ‘If I’d known this would happen, I wouldn’t have gone through with it’. But no one knew in advance that they would end up in the worst inflicted group of CIPN patients.” [participant 5]

Quote 134 - “So, what happened to this man?” [researcher] - “Well, he passed away…” [participant 5]
